# Selection of a Mimotope Peptide of *S*-adenosyl-l-homocysteine and Its Application in Immunoassays

**DOI:** 10.3390/molecules181013020

**Published:** 2013-10-18

**Authors:** Chun Wu, George Tzertzinis

**Affiliations:** 1 Health Research Institute, AIST, 1-8-31 Midorigaoka, Ikeda 563-8577, Osaka, Japan; 2 New England Biolabs, 240 County Road, Ipswich, MA 01938, USA

**Keywords:** homocysteine, competitive assay, phage display, monoclonal antibody, clinical diagnostics

## Abstract

A competitive immunoassay for *S*-adenosyl-l-homocysteine (SAH) has been used in the clinical test for homocysteine via an enzymatic conversion reaction. Since *S*-adenosyl-l-homocysteine is a relatively unstable compound, we have used peptide library phage display to select a new mimotope peptide that interacts with the anti-SAH antibody. By immobilizing the synthetic peptide on solid phase as a competitive surrogate for SAH, we demonstrate its utility in a competitive ELISA assay. The linear range of the assay for SAH was 0.4–6.4 µM, in good correlation to the conventional assay using an SAH-conjugated plate. Our results show that the mimotope peptide has potential to substitute for SAH in immunoassays.

## 1. Introduction

**Figure 1:**
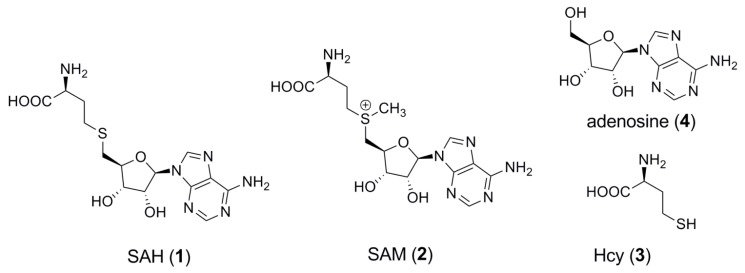
Chemical structures of SAH, SAM, Hcy and adenosine.

The chemical compound *S*-adenosyl-L-homocysteine (**1**, SAH, Figure 1) is an intermediate in methionine metabolism, which is observed in all organisms from bacteria to humans. It is produced by all *S*-adenosyl-L-methionine (**2**, SAM, Figure 1)-dependent enzymatic methylation reactions via SAM demethylation, and followed by conversion to homocysteine (**3**, Hcy, Figure 1) and adenosine (**4**, Figure 1) by the enzyme SAH hydrolase. Because SAM, SAH and Hcy are essential compounds in methionine metabolic pathways, the concentration of these compounds in plasma and intracellularly are important predictors of cellular methylation potential and metabolic alterations, and are associated with specific gene activity or age-related diseases. The analysis of these three metabolites in blood can be achieved by HPLC and various detection methods, such as UV-visible absorption spectrometry, coulometry or mass spectrometry have been reported [1,2,3]. However, HPLC analysis coupled with spectrometry requires time-consuming procedures and expensive instruments, making it unsuitable for routine clinical analyses. A solid-phase competitive enzyme-linked immunosorbent assay (ELISA) with a monoclonal antibody allows a low-cost high-throughput analysis of small molecules. A monoclonal antibody against SAH is commercially available. Due to the fact that SAM has a similar structure as SAH differing only by a methyl group, SAM could be an interfering compound in SAH-based immunoassays. Nevertheless, a rapid and precise immunoassay for quantification of Hcy with a monoclonal antibody against SAH has been reported [4]. This immunoassay is based on enzymatic conversion of Hcy to SAH with SAH hydrolase, followed by quantification of SAH by a competitive enzyme-linked immunoassay in a multi-well plate coated with SAH-conjugated BSA. This ELISA kit is commercially available from Axis/Abbot. This assay has the drawback that it relies on an unstable SAH-BSA conjugate.

SAH, a thioether-containing compound, is slowly oxidized in solution as well as in solid phase by molecular oxygen to yield the corresponding sulfoxide. The anti-oxidant thiodiglycol, (an intermediate for chemical weapon manufacture), has been used to protect against oxidation of SAH in solution [5]. In order to discover a substitute for the unstable SAH-BSA conjugate used in the immunoassay, we used phage display to discover a mimotope for SAH. 

Phage peptide libraries have been demonstrated to yield mimotope peptides for carbohydrates [6], fluorescent dyes [7], and low molecular weight toxins [8]. Furthermore a mimotope obtained from phage display has been recently demonstrated to substitute for aflatoxin in the corresponding immunoassay [9]. Therefore, we performed biopanning of phage display libraries using a specific antibody against SAH.

By immobilizing the synthetic peptide identified from screening phage display libraries on plates via biotin-streptavidin interaction, we demonstrated a competitive ELISA assay for SAH. The linear range of SAH in this assay was 0.4–6.4 µM, showing a good correlation to the conventional ELISA assay using SAH directly conjugated on a plate. Our results show that the mimotope peptide has a potential to substitute for SAH in this immunoassay.

## 2. Results and Discussion

### 2.1. Panning-Elution Selection of Specific Phage

Two commercially available phage display peptide libraries Ph.D.-7 and Ph.D.-C7C were used for the biopanning experiment. The Ph.D.7 library displays a linear heptapeptide, whereas the Ph.D.-C7C displays a loop-constrained heptapeptide formed by a pair of flanking disulfide linked cysteine residues. Although many mimotope peptides for protein antigens have been isolated with such libraries, only a few peptides that mimic a small molecule have been selected. The most successful example is a biotin mimic peptide, which interacts with streptavidin [10]. This is a first attempt to find a new peptide sequence that mimics SAH in an immunoassay. We did three rounds of panning-elution selection with the Ph.D.7 library. After three rounds of panning against the SAH-antibody, an increase in titer and a remarkable enrichment of a specific phage was achieved. To determine the affinity of the selected phage from the third-round, we randomly isolated and amplified eight phage clones by infection of *E. coli* ER2738. Six of these clones showed significant binding to the SAH antibody which was competitively inhibited by SAH, whereas two showed no significant binding. This strongly suggested that these six phage clones bound the antigen binding site of the SAH antibody. We amplified all selected phage clones by PCR and specific primers for the displayed peptide region. DNA agarose gel electrophoresis revealed that the two non-binding clones had no insert. The peptide sequence of the six binding phage clones was determined by DNA sequencing. Surprisingly all these phages had the same DNA sequence, which encoded the displayed peptide sequence FSDHWVN. On the other hand, following the same biopanning procedure with the Ph.D.-C7C library, no specific consensus sequence was enriched. (Table 1). This result suggests that the disulfide loop-constrained library does not contain any clones that bind tightly antibody.

**Table 1:** Peptide sequences obtained from the Ph.D.-7 and Ph.D.-C7C libraries.

*Ph.D-7*	*Ph.D.-C7C*
FSDHWVN	CVQMPAHSC
FSDHWVN	CPNSTHRNC
FSDHWVN	CMHTHSRMC
FSDHWVN	CNTGSPYEC
FSDHWVN	CFSGMSTDC
FSDHWVN	CDASRPATC
--	CRGATPMSC
--	CSEGLLNTC

### 2.2. Use of the Discovered Mimotope Peptide in Immunoassay of SAH

**Figure 2:**
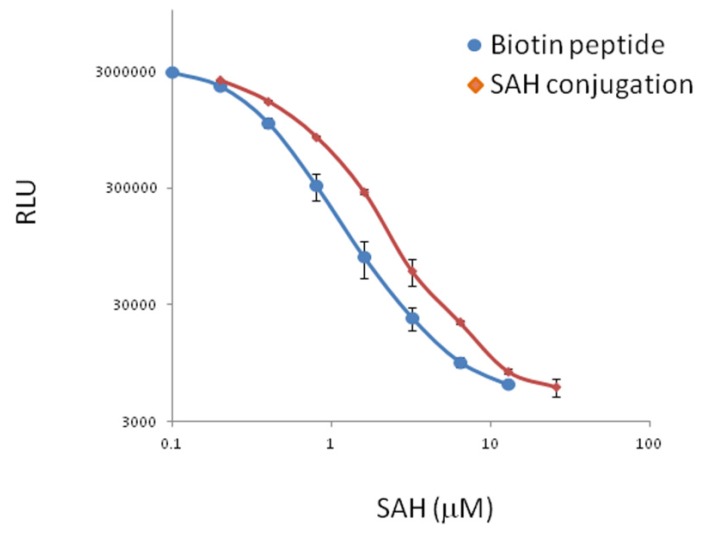
SAH calibration curves in competitive immunoassay using the biotin-mimotope peptide or SAH conjugated to the assay plate. Chemiluminescent detection was achieved with an HRP-conjugated secondary antibody (n = 3).

To determine whether the sequence obtained from the phage bound to the anti-SAH antibody is independent of the phage structure context, we synthesized the peptide (FSDHWVNGGGS-Biotin). The immobilized peptide *via* biotin-streptavidin interaction showed specific binding to the antibody against SAH. Furthermore, free SAH showed competitive inhibition of the peptide binding over a wide range of concentration (0.1~12.8 µM). This data is in good correlation with conventional ELISA assays using the SAH directly conjugated to the plate (Figure 2). This data indicates that the discovered peptide alone is sufficient for binding to the antigen binding site of the antibody, independent of the phage structure context. In the previously reported ELISA assay, a concentration range of 2–50 µM SAH was used in the calibration curve [4]. The linear range of SAH in the competitive assay using chemiluminescent detection with our peptide is 0.4–6.4 µM. This result demonstrates the potential for using this peptide to detect a lower range of concentrations of SAH, resulting in more sensitive ELISA type immunoassays. 

**Figure 3:**
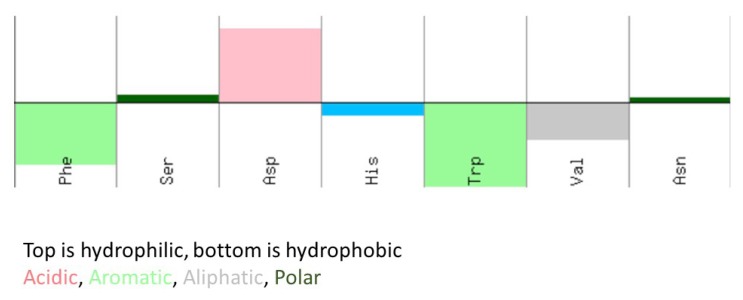
Hopp and Woods hydropathy scale of the selected peptide.

*S*-Adenosyl-l-homocysteine (SAH) has a hydrophobic adenine ring and a hydrophilic homocysteine moiety. The *p*Ka value of adenine ring on the N1 position was reported to be 4.1 [11] Thus the adenine ring is not charged at the neutral pH of the assay. SAH was conjugated to the plate via the amino group of the homocysteine moiety. This orientation suggests that hydrophobic interactions with the adenine moiety is essential for the binding of the antibody. The *p*I value of the mimotope peptide was calculated to be about 4.9, so the net charge of this peptide at pH7 is slightly negative. However the peptide has 43% hydrophobic and 29% hydrophilic property (Figure 3). It is likely that the hydrophobic moieties of the residues of F and W contribute the hydrophobic interaction between the peptide and the anti-SAH antibody. The detailed hydrogen bonding, however, between the peptide and the antibody is unknown. The molecular structure analysis of the complex between this peptide and the antibody is currently in progress.

## 3. Experimental

### 3.1. Materials

Protein A magnetic Beads, protein G magnetic beads, the Ph.D.-7 and Ph.D.-C7C Phage Display Library Kits including the −28 gIII and −96 gIII sequencing primers, and *E. coli* ER2738 host strain were from New England Biolabs (Ipswich, MA, USA). The anti-SAH rabbit MAb (ab111903) and the HRP-conjugated anti-rabbit IgG goat polyclonal antibody were purchased from Abcam (Cambridge, MA, USA). The chemiluminescent substrate for HRP, maleic anhydride-activated plates and streptavidin-coated plates were purchased from Pierce (Rockford, IL, USA). The synthetic SAH and other reagents were purchased from Sigma-Aldrich (St. Louis, MO, USA). 

### 3.2. Selection of anti-SAH MAb Binding Peptides from Phage Library

Two micro liters of the original phage libraries (Ph.D.-7 or Ph.D.-C7C) were diluted to 200 µL with TBST (TBS + 0.1% [v/v] Tween-80). The SAH antibody (300 ng) was added to the TBST solution. The mixture was incubated at room temperature for 20 minutes with gentle agitation. The antibody-bound phages were then captured with Protein A or Protein G magnetic beads. Protein G magnetic beads were used in the first and third rounds. Protein A magnetic beads were used in the second round. The magnetic beads were washed 10X with TBST to remove non-binders. The bound phage was eluted with 0.2 M glycine–HCl (pH 2.2) in 1 mg/mL BSA. The eluate was transferred into a micro centrifuge tube, and neutralized with 15 µL of 1 M Tris–HCl (pH 9.1). The eluted phage was titered onto LB/IPTG/Xgal plates to determine the concentration of phage in the eluate. Eluted phage was amplified by incubating at 37 °C with vigorous shaking with an early log phase ER2738 culture for 4.5 h. Purification was performed by first centrifuging the culture at 12,000 g at 4 °C to pellet the ER2738 cells. The supernatant was removed and mixed with 1/6 equivalent volume of PEG/NaCl (20%w/v) polyethylene glycol-8000, 2.5 M NaCl). After cooling overnight at 4 °C, the resulting suspension was again centrifuged at 9,000 rpm. The resulting pellet was collected and resuspended in 1 mL TBS. Further centrifugation pelleted residual cells, and 80% of the suspension was collected. One-sixth of equivalent volume of PEG/NaCl was added and the solution incubated on ice for 1 h. The suspension was micro-centrifuged at 4 °C for 20 min and the supernatant removed and discarded. The pellet was resuspended in 200 µL TBS. The concentration of the phage was determined by titering onto LB/IPTG/Xgal plates. The process was repeated for two additional panning rounds using the obtained eluate. The final eluate was plated onto a LB/IPTG/Xgal plate to yield approximately 50 individual clones. Twenty clones from the plate were chosen at random and the displayed sequence was amplified by PCR with the −96 gIII sequencing and M13 extension primers (E8101S) and submitted to DNA sequencing with the −96 gIII sequencing primer. The C-terminally biotinylated peptide (FSDHWVNGGGS-Biotin) was synthesized in-house by the NEB Organic Synthesis Facility (Beverly, MA, USA). Peptide hydropathy was plotted with the tool provided at the website “pepcalc.com”. 

### 3.3. Use of the Mimotope Peptide for Immunoassay of SAH

The synthetic peptide was dissolved in DMSO and then diluted with PBS buffer containing 0.1 M potassium phosphate (pH 7.2) and 0.15 M NaCl to a final concentration of 1 µg/ml. One hundred µl of this solution was then added to each well of a 96-well streptavidin plate and incubated at room temperature for 1 h. After the plate was washed three times with washing buffer (20 mM Tris-HCl (pH7.8)/0.1%Tween/0.9% NaCl), we added the anti-SAH antibody in PBS buffer/0.5% BSA and a serial dilution of SAH standard ranging from 0.1 to 12.8 µM to each well, and then incubated them at room temperature for 2 h. After the wells were washed three times with washing buffer, HRP-conjugated anti-rabbit IgG antibody was added to each well, and incubation was carried out for another 2 h at room temperature. After all wells were washed as above and briefly dried, luminescence from bound antibody was measured by adding chemiluminescent substrate to each well. For comparison purposes, we directly conjugated SAH directly to maleic anhydride-activated plates (Pierce) according to the instructions from the manufacturer. A serial dilution of SAH standard ranging from 0.2 to 25.6 µM was used in the same competitive format assay

## 4. Conclusions

We identified a phage clone encoding a mimotope for an anti-SAH monoclonal antibody by screening a phage display library Ph.D.7. The synthetic peptide (FSDHWVNGGGS-Biotin) specifically competes with SAH for binding to the same antibody. Our results demonstrate the potential for using this peptide in sensitive SAH detection immunoassays.
